# Weakly Supervised Gland Segmentation Based on Hierarchical Attention Fusion and Pixel Affinity Learning

**DOI:** 10.3390/bioengineering12090992

**Published:** 2025-09-18

**Authors:** Yanli Liu, Mengchen Lin, Xiaoqian Sang, Guidong Bao, Yunfeng Wu

**Affiliations:** School of Informatics, Xiamen University, 422 Si Ming South Road, Xiamen 361005, China; liuyanli@stu.xmu.edu.cn (Y.L.); 23320201154003@stu.xmu.edu.cn (M.L.); 23320201154016@stu.xmu.edu.cn (X.S.); 23320201153974@stu.xmu.edu.cn (G.B.)

**Keywords:** gland segmentation, weakly supervised learning, multi-level attention mechanism, affinity learning

## Abstract

Precise segmentation of glands in histopathological images is essential for the diagnosis of colorectal cancer, as the changes in gland morphology are associated with pathological progression. Conventional computer-assisted methods rely on dense pixel-level annotations, which are costly and labor-intensive to obtain. The present study proposes a two-stage weakly supervised segmentation framework named Multi-Level Attention and Affinity (MAA). The MAA framework utilizes the image-level labels and combines the Multi-Level Attention Fusion (MAF) and Affinity Refinement (AR) modules. The MAF module extracts the hierarchical features from multiple transformer layers to grasp global semantic context, and generates more comprehensive initial class activation maps. By modeling inter-pixel semantic consistency, the AR module refines pseudo-labels, which can sharpen the boundary delineation and reduce label noise. The experiments on the GlaS dataset showed that the proposed MAA framework achieves the Intersection over Union (IoU) of 81.99% and Dice coefficient of 90.10%, which outperformed the state-of-the-art Online Easy Example Mining (OEEM) method with an improvement of 4.43% in IoU. Such experimental results demonstrated the effectiveness of integrating hierarchical attention mechanisms with affinity-guided refinement for annotation-efficient and robust gland segmentation.

## 1. Introduction

Colorectal cancer is a prevalent malignant tumor that originates in the colon or rectum. It is one of the top causes of cancer-related mortality globally. In clinical practice, diagnostic approaches for colorectal cancer include computed tomography, magnetic resonance imaging, colonoscopy, and biopsy [1,2]. Histopathological analysis of biopsy samples is particularly regarded as the gold standard for diagnosis and tumor grading. Gland segmentation in histopathological images is of particular importance in colorectal adenocarcinoma, as morphological changes in glandular structures are strongly correlated with tumor progression [3]. Accurate delineation of gland boundaries enables precise characterization of tissue architecture and supports effective cancer staging.

In clinical practice, gland segmentation and tumor grading are performed manually by pathologists based on their domain expertise. However, such a process is labor-intensive, time-consuming, and subject to intra- and inter-observer variability. The complex structure of glandular tissues further increases the difficulty, as gland boundaries are often blurred or indistinguishable from the background due to staining inconsistencies, overlapping cells, and densely packed tissue structures. Such challenges emphasize the pressing necessity of automated and objective gland segmentation tools. Recent advances in computer-aided segmentation techniques have greatly improved the semantic consistency and efficiency of pathology workflows [4,5]. The emerging deep-learning tools have revolutionized digital pathology. Convolutional neural networks (CNNs), fully convolutional networks (FCNs), and Transformer-based models have been widely utilized for medical image segmentation applications [6,7,8,9,10].

Fully supervised deep-learning methods have achieved remarkable success in various segmentation tasks by leveraging large-scale pixel-level annotations. Ronneberger et al. [11] proposed the U-Net as a CNN-based model designed for biomedical image segmentation. Its encoder–decoder structure and skip connections can preserve spatial resolution during upsampling. Chen et al. [12] developed the DeepLabV3+, an FCN-based framework that integrates atrous spatial pyramid pooling (ASPP) and an improved encoder–decoder structure. Recently, the UNETR proposed by Hatamizadeh et al. [13] used a transformer-based architecture to directly model long-range dependencies in volumetric medical data, and could achieve competitive results in 3D medical image segmentation tasks [3]. These approaches may exhibit effectiveness, but they still rely heavily on dense, pixel-level annotations. Moreover, such expert-labeled annotations are laborious to outline histopathological contexts associated with pathology, and difficult to standardize in clinical practice as well.

Weakly supervised image segmentation (WSIS) [14] has attracted extensive attentions for its advantage of reducing annotation burdens by learning from coarse labels such as scribbles [15], bounding boxes [16], points [17], or image-level tags [18]. Image-level labels are particularly appealing because they can be efficiently derived from routine clinical diagnoses without extra annotation efforts. In the follow-up study of Zhou et al. [19], the Class activation maps (CAMs) were used to extract the localization cues with global average pooling, and align the image-level labels with pixel-wise segmentation. Improvements of the CAMs include the suppressing dominant regions [20], accumulating attention across training iterations [21], and mining multi-image semantic cues [22]. However, the sparse supervision signal provided by such coarse labels commonly leads to incomplete or inaccurate localization, especially in gland segmentation tasks that require essential fine-grained morphological details. Such challenges motivate the advance in novel WSIS frameworks that can accomplish accurate segmentation with limited supervision.

A variety of WSIS techniques under sparse annotations have been developed in recent years. For instance, Wang et al. [23] developed the self-supervised equivariant attention mechanism (SEAM) which enforces consistency between CAMs generated from transformed inputs. The Adv-CAM is able to generate the adversarial perturbations that expand object regions beyond the initially activated CAM regions, in order to recover the missing object parts ignored by sparse labels [24]. The SC-CAM may enhance weakly supervised segmentation by subcategory-aware learning, which guides the model to focus on the ambiguous regions caused by sparse labels [25]. These methods have achieved solid performance in natural image segmentation but do not explicitly account for the unique challenges of histopathological data.

Li et al. [26] proposed the Online Easy Example Mining (OEEM) as a weakly supervised framework for gland segmentation in histopathological images. Such a two-stage method reduces the noise in pseudo-labels by introducing a normalized loss function that emphasizes reliable supervision and downweighting noisy regions. The OEEM [26] is a two-stage weakly supervised framework specifically designed for gland segmentation in histopathology. It tackles the issue of visual homogeneity between glandular structures and background regions due to similar color characteristics. The OEEM applies a loss re-weighting strategy that suppresses noisy supervision and highlights high-confidence regions. The OEEM architecture may achieve the performance close to fully supervised methods by mainly improving robustness during the segmentation stage. Although the OEEM is currently prevailing for medical segmentation tasks, its effectiveness still remains limited due to potential incomplete activation of pseudo-labels in the CAM generation stage.

The WSIS methods reported in the previous studies still face several challenges for complex medical image segmentation tasks. Most approaches heavily rely on the CAMs generated from convolutional networks, which have limited the capability to obtain the long-range dependencies and hierarchical context. Such a limitation is particularly evident in histopathological gland segmentation, where the foreground–background contrast is subtle and the glandular structures often exhibit high morphological variability. The incomplete activations and ambiguous boundaries in initial localization maps result in unreliable pseudo-labels with noise and fragmentation. A more effective framework is urgently demanded to improve both semantic representation and pseudo-label quality under weak supervision.

In the present study, we propose a novel two-stage weakly supervised segmentation framework, termed Multi-Level Attention and Affinity (MAA), for the segmentation of glands in histopathological images. The MAA is able to enhance the pseudo-label quality and boundary precision with only image-level supervision. It consists of two core components, i.e., the Multi-Level Attention Fusion (MAF) module and the Affinity Refinement (AR) module. The MAF module aggregates hierarchical Transformer features to capture long-range semantic dependencies. The AR module refines the initial pseudo-labels by modeling the inter-pixel relationships and suppressing noisy activations. The MAA combines the multi-scale attention and semantic consistency to handle faint gland-background differences and diverse gland shapes, which leads to more reliable weakly supervised segmentation. The major contributions of the present work are summarized as follows.

- A novel weakly supervised segmentation framework named MAA is developed, which leverages hierarchical attention features and pixel-level affinity learning to enhance pseudo-label quality under image-level supervision.
- A MAF module is proposed to effectively capture global semantic context and resolve incomplete activations in traditional CAM-based methods.
- An AR module is designed to exploit semantic consistency between pixels, improving the reliability of pseudo-labels, particularly in ambiguous regions with blurred gland-background boundaries.
- Extensive experiments on the GlaS dataset demonstrate that the proposed MAA framework achieves superior performance compared to several state-of-the-art weakly supervised segmentation approaches. Ablation studies further validate the respective contributions of the MAF and AR modules to the proposed framework.

## 2. Materials

Our experiments were carried out based on the GlaS dataset of the Histology Image Challenge [27]. The dataset includes a total of 165 whole-slide images from 16 Hematoxylin and Eosin (H & E) stained histological sections, which are associated with T3 or T42 stage colorectal adenocarcinoma. According to Sirinukunwattana et al. [27], the whole-slide images were digitalized from the 16 histological sections by using a Zeiss Slide Scanner (Model: MIRAX MIDI, resolution: 0.465 µm per pixel, Carl Zeiss Microimaging GmbH, Jena, Germany). The whole-slide images were rescaled into a resolution of 0.620 µm per pixel (equivalent to 20× objective magnification). The whole-slide image sizes in the dataset are listed in Table 1. For more details about the image data acquisition, readers are referred to the previous study of Sirinukunwattana et al. [27].

The GlaS dataset of 165 whole-slide images was divided into 85 images for training and the remaining 80 images for testing in our experiments. Each image of the GlaS dataset contains different glandular areas and backgrounds, so that it is difficult to assign a single glandular-level label to one image. In the preprocessing procedure, we cropped the original images of the training set into patches of 112×112 pixels with an overlap interval of 56 pixels. For instance, an original image with size of W×H could be cropped into *n* small images with the mathematical expression as

(1) $$\begin{array}{c} n=\left(\lceil \frac{W-112}{56}\rceil +i\right)\times \left(\lceil \frac{H-112}{56}\rceil +j\right),\\ i=\left\{\begin{array}{cc} 0, & \mathrm{if}\;W\;\mathrm{mod}\;112=0\\ 1, & \mathrm{if}\;W\;\mathrm{mod}\;112\neq 0, \end{array}\right.\;j=\left\{\begin{array}{cc} 0, & \mathrm{if}\;H\;\mathrm{mod}\;112=0\\ 1, & \mathrm{if}\;H\;\mathrm{mod}\;112\neq 0, \end{array}\right. \end{array}$$

where the sign ⌈·⌉ represents a round up to the nearest integer.

**Figure 1:**
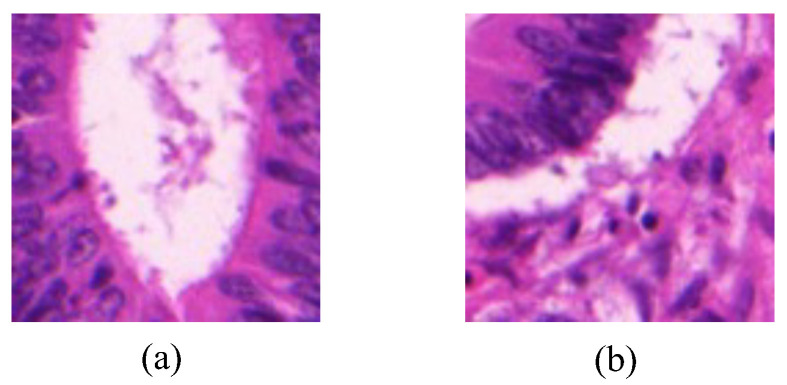
Examples of gland images with image-level labels. Subfigure (**a**) is annotated with the label [1, 0], indicating an image with a gland but no background. Subfigure (**b**) is annotated with the label [1, 1], indicating an image with both a gland and background.

Image-level labels is composed of a glandular label and a background label, both of which are represented with binary values. The glandular label in one image is assigned with 1 if there exhibits a gland area, or is assigned with 0 if no gland area. The background label is assigned with 1 if the image contains the background area, or otherwise is assigned with 0. Figure 1 shows examples of two gland images with different image-level labels.

After the preprocessing procedure, we converted 85 whole-slide images into a total of 9703 cropped images as the training set, with the annotated image-level labels for each cropped image according to the original ground truth provided by the dataset. For the purposes of visualization and evaluation of segmentation performance, all the cropped images were merged to rebuild their original whole-slide images.

## 3. Proposed Multi-Level Attention and Affinity Method

### 3.1. Two-Stage Architecture for Weakly Supervised Gland Segmentation

**Figure 2:**
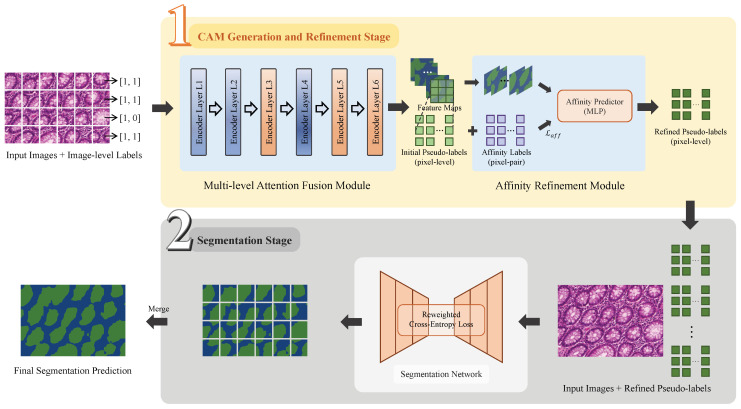
Framework of the two-stage MAA models for weakly supervised gland segmentation. Stage 1: CAM Generation and Refinement; Stage 2: Segmentation. The gland regions are displayed in green color.

As illustrated in Figure 2, the proposed MAA method establishes a two-stage network architecture to accomplish the weakly supervised gland segmentation. The framework includes a class activation map (CAM) generation and refinement stage and a segmentation stage. In the CAM generation and refinement stage, the CAMs are generated and refined to form pseudo-labels under weak supervision. These pseudo-labels are used as surrogate annotations in the Segmentation stage, which support a fully supervised training for the final segmentation prediction.

- In the CAM generation and refinement stage, the MAF module takes gland images divided into multiple cropped patches and their image-level labels as input. The multi-level features extracted by the Transformer encoder layers are fused to generate initial class activation maps (CAMs), which are optimized under image-level supervision without using ground-truth segmentation masks. These CAMs serve as initial pseudo-labels, and an AR module further refines their spatial coherence before they are forwarded to the Segmentation stage.
- In the segmentation stage, the PSPNet is employed as the backbone and trained with the refined pseudo-labels as supervision. Given the presence of inevitable noise in these pseudo-labels, a normalized loss function is then introduced to emphasize the informative regions when suppressing some unreliable areas. Such a strategy mitigates the negative impact of label noise, and improves the segmentation performance under the pseudo-supervised training.

### 3.2. Multi-Level Attention Fusion Module

**Figure 3:**
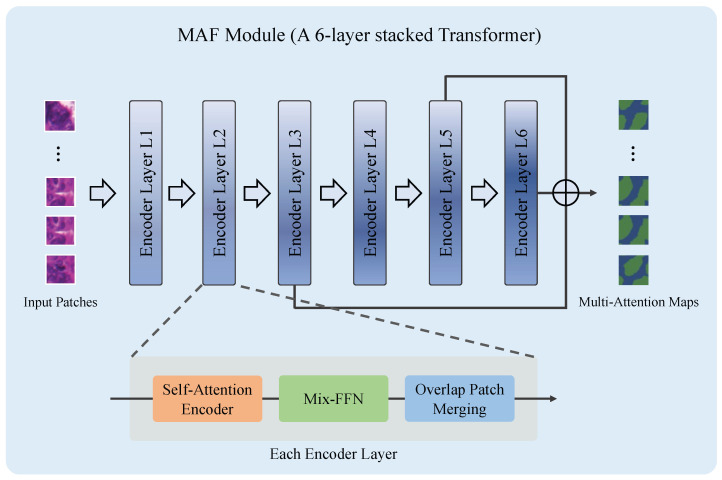
Architecture of the Multi-Level Attention Fusion (MAF) module. The MAF consists of a single Transformer composed of six stacked encoder layers. Each encoder layer contains three sub-modules: a self-attention block, a Mix Feed-Forward Network (Mix-FFN) block, and an overlapping patch merging block. The gland regions of the multi-attention maps are displayed in green color.

The MAF module is the core of the CAM generation and refinement stage. As shown in Figure 3, the MAF module is implemented as a single, stacked Transformer encoder composed of six sequential encoder layers to generate the hierarchical multi-resolution feature maps from the input images. Each encoder layer consists of three sub-modules: a self-attention encoder, a Mix Feed-Forward Network (Mix-FFN), and an overlapping patch merging unit.

The self-attention encoder captures the global contextual relationships between image patches by computing the pairwise attention weights, enabling the network to model the long-range dependencies and inter-region correlations. Inside the Mix-FFN, a 3×3 convolution is embedded in the feed-forward path to preserve local neighborhood cues and strengthen the position-related representations.

After the Mix-FFN, the overlapping patches are merged to downsample the spatial resolution and increase the channel depth. For an input image of size H×W×3, the hierarchical feature maps produced by the *i*-th layer can be written as:

(2) $$F_{i}\in R^{\frac{H}{2^{i+1}}\times \frac{W}{2^{i+1}}\times C_{i}},\;i\in \left\{1,2,3,4,5,6\right\}.$$

Here, $F_{i}$ denotes the feature map produced by the *i*-th layer, which is a real-valued tensor of size $\left(H/2^{i+1}\right)\times \left(W/2^{i+1}\right)\times C_{i}$. *H* and *W* are the height and width of the input image, respectively, $C_{i}$ indicates the channel depth at the *i*-th layer, and *i* is the layer index, starting from 1.

During the experiments, we repeated the first two sub-modules twice in each layer, in order to improve the representational capacity. The combined operation of these three components enables each encoder layer to preserve and enrich the spatial information across scales, even without explicit positional encoding.

The encoder layer produces hierarchical feature maps $F_{i}$ with progressively lower spatial resolution and higher semantic abstraction [28]. Each layer receives the output of the previous one, and produces a hierarchical representation with the resolutions of 1/4, 1/8, 1/16, 1/32, 1/64, and 1/128 of the original image. We fuse features from the 3rd, 5th, and 6th layers, which respectively emphasize coarse gland localization, structural completeness, and boundary refinement. The choice of these layers is based on visual inspection of the fused outputs and support from prior work on hierarchical multi-level feature fusion in Transformers [28]. To fuse the heterogeneous feature maps, each $F_{i}$ is first resized to a common spatial resolution via bilinear upsampling and aligned in channel dimension using a 1×1 projection:

(3) $${\tilde{F}}_{i}={\mathrm{Proj}}_{1\times 1}\left(\mathrm{Upsampling}\left(F_{i};S\right)\right),\;i\in \left\{3,5,6\right\},$$

where *S* denotes the target resolution. The multi-level representation is then obtained by element-wise summation:

(4) $$F_{\mathrm{multi}}=\sum_{i\in \{3,5,6\}}{\tilde{F}}_{i}.$$

This fused representation is subsequently passed through a projection head to generate the initial CAM, which serves as the initial pseudo-label providing surrogate supervision for downstream steps.

**Figure 4:**
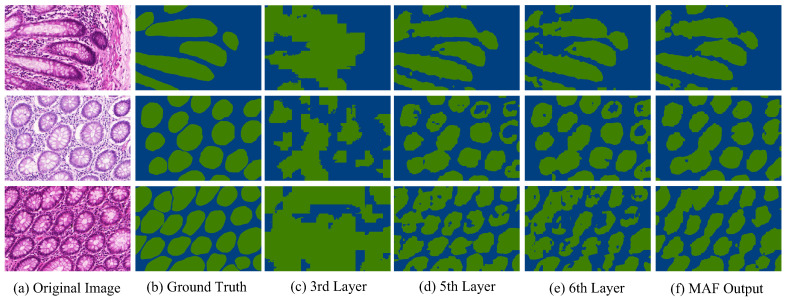
Visualization of selected attention layer outputs and the final pseudo-label generated by the MAF module, shown alongside the original image and corresponding ground truth. The gland regions are displayed in green color.

As illustrated in Figure 4, visualization comparisons showed that the third layer captures coarse localization of glandular regions, while the fifth and sixth layers provide increasingly refined boundary details. The combination of attention outputs of these three layers produced the pseudo-labels that could better approximate the ground truth and offer more reliable guidance for downstream segmentation. Compared with the standard class activation maps (CAMs), the MAF-based strategy generated the initial pseudo-labels with higher spatial precision and reduced noise.

### 3.3. Affinity Refinement Module

To further enhance the quality of initial pseudo-labels produced by the MAF module, a trainable affinity-guided refinement module is introduced to refine the coarse delineation of glandular boundaries, and also enhance the label precision by enforcing the semantic consistency at pixel level. It learns the semantic affinity relationships between pixels using the self-attention features generated by the Transformer encoder. Specifically, a multi-layer perceptron (MLP) encodes the multi-level attention maps produced by the MAF module, and generates the affinity scores that reflect the likelihood between two pixels in the same semantic region. In our framework, the MLP is implemented with three fully connected layers. Its input layer is formed by concatenating the fused multi-level feature representations with the embedded initial pseudo-labels, and each node in the output layer produces a single scalar affinity score for each pixel pair. This design enables the network to efficiently transform both contextual features and coarse label guidance into semantic affinity predictions, which are then used to refine the pseudo-labels.

The affinity labels, denoted as $Y_{p}^{i,j}$, indicate whether the pixel pair (i,j) belong to the same semantic region and are used to supervise the training of the affinity module. These labels are derived by combining the initial pseudo-labels produced by the MAF module with local consistency information extracted from the original image using the Pixel-Adaptive Refinement (PAR) method [29].

(5) $$Y_{p}^{i,j}=\left\{\begin{array}{cc} argmax(L_{\mathrm{init}}^{i,j}), & \mathrm{if}\;\max\left(L_{\mathrm{init}}^{i,j}\right)\geq \beta_{h}\\ 0, & \mathrm{if}\;\max\left(L_{\mathrm{init}}^{i,j}\right)\leq \beta_{l}\\ 255, & \mathrm{otherwise} \end{array}\right.$$

where $L_{\mathrm{init}}$ represents the initial pseudo-labels, and $\beta_{l}$ and $\beta_{h}$ are thresholds satisfying $0<\beta_{l}<\beta_{h}<1$ to separate foreground, background, and uncertain regions. In our experiments, we set $\beta_{l}=0.3$ and $\beta_{h}=0.6$. Here, 0 and 255 denote the background and uncertain categories, respectively, and argmax(·) returns the foreground class with the highest activation.

The PAR mechanism leverages both the RGB and positional information to identify the reliable pixel-pair relationships, from which positive and negative sets (denoted as $R^{+}$ and $R^{-}$) are constructed for supervision. Only pixel pairs within a local neighborhood of radius *r* are considered, where r=8. Pairs beyond this spatial extent are disregarded to ensure that the affinity estimation remains spatially coherent and reliable.

The affinity loss function $L_{\mathrm{aff}}$ guides the network to learn the high-confidence affinity representations, so that the self-attention mechanism can capture more comprehensive and coherent glandular structures.

(6) $$L_{\mathrm{aff}}=\frac{1}{N^{-}}\sum_{\left(i,j\right)\in R^{-}}\left(1-\mathrm{sigmoid}(A^{ij})\right)+\frac{1}{N^{+}}\sum_{\left(i,j\right)\in R^{+}}\mathrm{sigmoid}\left(A^{ij}\right)$$

where $A^{ij}$ denotes the predicted semantic affinity score for pixel pair (i,j). $N^{+}$ and $N^{-}$ represent the number of positive and negative samples, respectively.

**Figure 5:**
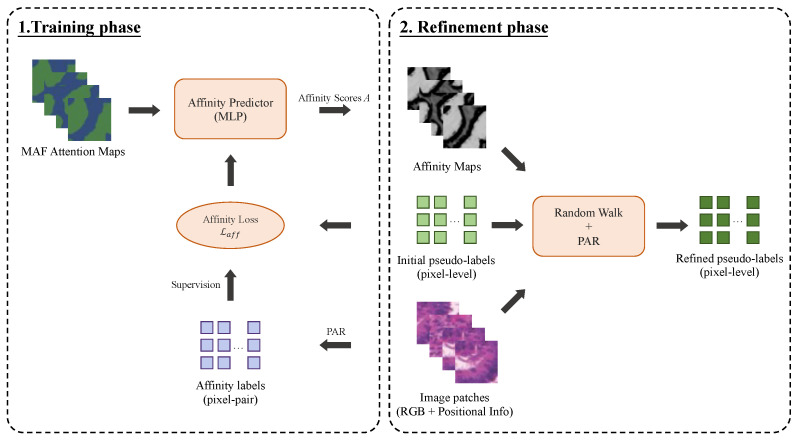
Workflow of the affinity refinement module. In the training phase, the gland regions of the MAF attention maps are displayed in green color.

As illustrated in Figure 5, the AR module is trained iteratively with the affinity loss, to progressively improve affinity predictions. After training, the generated semantic affinity maps are combined with the initial pseudo-labels to produce the final pseudo-labels through a two-step process involving the PAR [29] and random walk propagation [30]. In this phase, the PAR is again implemented to adaptively smooth the initial pseudo-labels based on the learned affinity and image content, followed by a random walk algorithm to propagate semantic consistency across the entire image.

Mechanistically, the affinity refinement module mitigates local noise by leveraging pixel-pair affinities derived from feature similarity and spatial proximity. Isolated noisy spikes in the initial CAMs tend to have low affinities with their neighbors and are thus suppressed during label propagation, while coherent glandular regions exhibit high mutual affinities, leading to enhanced structural consistency and smoother pseudo-labels. Such a combination sharpens boundary transitions and corrects local inconsistencies, to produce the refined pseudo-labels with improved boundary delineation and semantic coherence. Compared with the initial labels, the refined pseudo-labels show a higher spatial accuracy, clearer boundaries, and reduced noise. These high-quality labels are then used to supervise the following segmentation stage and provide a more reliable foundation for fully supervised training.

### 3.4. Loss Function Design in Segmentation Stage

In the segmentation stage, the network performs the dense pixel-wise classification under a fully supervised learning framework. A Pyramid Scene Parsing Network (PSPNet) [31] with a pretrained ResNet backbone is employed to extract the multi-scale contextual features and support accurate semantic segmentation. The PSPNet is trained with pseudo-labels generated in the previous stage. These labels commonly involve noise, particularly in boundary areas or regions with visual ambiguity, which might complicate learning. In order to eliminate the impact of noise, a normalized loss function based on the OEEM [26] is considered.

The OEEM-based loss function adjusts the pixel-wise loss, by emphasizing informative and reliable supervision. This re-weighting strategy modifies the standard cross-entropy loss $L_{\mathrm{ce}}$ by assigning higher weights to low-loss pixels and suppressing the influence of noisy, high-loss samples. Therefore, clean pixels with lower loss would receive more learning attention. Such a mechanism enables the segmentation network to gradually refine its predictions and suppress error accumulation, particularly in the regions with pseudo-label noise or inter-gland homogeneity. In each training batch, the model prioritizes accurate supervision and gradually adapts to harder examples, which improves robustness to pseudo-label imperfections and enhances segmentation performance.

The normalized loss function *L* is defined as follows:

(7) $$\left.L=\left\{\begin{array}{cc} L_{\mathrm{wce}}, & n>1\\ L_{\mathrm{ce}}, & n=1 \end{array}\right.\right.,$$

(8) $$L_{\mathrm{wce}}=-\sum_{j=0}^{W}\sum_{i=0}^{H}w_{i,j}\cdot \log\frac{\exp\left({\tilde{X}}_{Y_{i,j},i,j}\right)}{\sum_{k=1}^{C_{i}}\left(\exp\left({\tilde{X}}_{k,i,j}\right)\right)},$$

(9) $$w=\frac{\mathrm{softmax}(-L)}{\mathrm{mean}(\mathrm{softmax}(-L))},$$

where $\tilde{X}$ denotes the predicted segmentation map, *Y* is the pseudo ground truth label, *w* is the dynamically computed weight map, and $L_{\mathrm{wce}}$ is the weighted pixel-wise cross-entropy loss, respectively. In the case where an image just contains a single class, the standard cross-entropy is then applied without re-weighting.

## 4. Experiments and Result Analysis

### 4.1. Experiments

**Figure 6:**
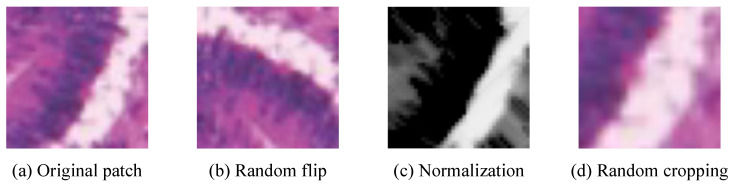
Visualizations of a gland image processed with data augmentation.

The workflow of our experiments contains three major components: data preprocessing, the CAM generation and refinement stage, and the segmentation stage. A data augmentation preprocessing procedure was utilized to increase data diversity and improve the generalization capability. The data augmentation techniques included the random horizontal and vertical flips (each with a probability of 0.5), a per-channel normalization using dataset-specific means and standard deviations, and a random cropping with crop ratios ranging from 0.7 to 1.0. Data augmentation visualizations of an example gland image are shown in Figure 6. All models were trained on a Linux server equipped with two NVIDIA GeForce RTX 3090 GPUs.

In the CAM generation and refinement stage, the network was trained using the stochastic gradient descent (SGD) optimizer with a polynomial decay schedule. The learning rate was controlled by the CosineAnnealingLR strategy, with an initial (and maximum) learning rate of 0.002 and a minimum learning rate of 0. The batch size was set to 16, and the model was trained for 200 epochs.

In the segmentation stage, the model training and evaluation were implemented using the MMSegmentation framework, an open-source semantic segmentation toolbox built on PyTorch v2.7. The segmentation network was optimized using the SGD algorithm with a fixed learning rate of 0.0005, a batch size of 32, and a total of 10,000 iterations.

### 4.2. Evaluation Metrics

The gland segmentation task is essentially a pixel-wise classification problem. The goal is to separate the glandular regions from background in histopathological images. In order to evaluate segmentation performance, two commonly used quantitative metrics, i.e., Intersection over Union (IoU) and Dice coefficient, were computed in the present study.

Let *X* denote the set of pixels belonging to predicted segmentation object (gland or background), and *Y* represent the set of pixels belonging to a ground truth object. The IoU and Dice coefficient are calculated as follows:

- Intersection over Union (IoU) characterizes the ratio of the overlap area between the predicted segmentation and the ground truth over the total combined area covered by both the prediction and the ground truth, which is calculated as [32]:

(10) $$\mathrm{IoU}\;=\frac{\left|X⋂Y\right|}{\left|X⋃Y\right|}=\frac{\left|X⋂Y\right|}{\left|X\right|+\left|Y\right|-\left|X⋂Y\right|}.$$

- Dice coefficient, also referred to as the Sørensen–Dice index, measures the similarity between two sets, which can be mathematically convertible from IoU as [33]:

(11) $$\mathrm{Dice}\;=\frac{2\;\left|X⋂Y\right|}{\left|X\right|+\left|Y\right|}=\frac{2\times \mathrm{IoU}}{(1+\mathrm{IoU})}.$$

The IoU and Dice both range from 0 to 1. These metrics were used to quantify the segmentation accuracy in identifying glandular structures, with higher scores indicating superior localization and boundary precision results.

### 4.3. Model Comparison

To compare the effectiveness of the proposed MAA framework with the state-of-the-art methods, we also implemented several representative weakly supervised semantic segmentation models, such as the SEAM, Adv-CAM, and SC-CAM, for the gland segmentation tasks. In addition, the OEEM was selected as a domain-specific baseline tailored for histopathological image segmentation.

As shown in Table 2, both the OEEM and MAA methods were able to achieve significantly better performance than the SEAM, Adv-CAM, and SC-CAM methods. The OEEM and MAA are domain-specific methods designed for histopathological image segmentation. The other three methods are originally developed for natural images and fail to address the particular challenges posed by medical data, such as subtle gland–background distinctions and high inter-class similarity. Moreover, both the OEEM and MAA methods utilized a two-stage framework that separates the CAM generation and final segmentation. Such an architecture enables the progressive refinement of supervision signals, which helps improve the segmentation accuracy.

**Figure 7:**
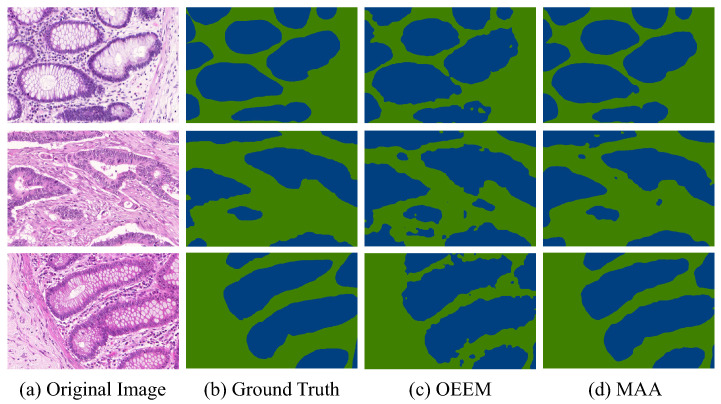
Visual comparison of segmentation results: (**a**) original image, (**b**) the ground truth, (**c**) the OEEM baseline, and (**d**) the proposed MAA method. The gland regions are displayed in blue color.

Compared with the OEEM, the MAF and AR modules enabled the MAA method to better capture global contextual information and improve pseudo-label precision, especially at gland boundaries. As reported in Table 2, the MAA surpasses the OEEM by 4.43% in IoU and 2.07% in Dice, achieving 81.99% and 90.10%, respectively. This improvement demonstrates the effectiveness of Transformer-based feature aggregation and affinity-guided supervision under weak supervision. Qualitative results in Figure 7 further confirm the superiority of the proposed method. The MAA produces more accurate and coherent segmentation maps than OEEM on representative samples from the GlaS testing set. Both methods detect glandular regions, but the MAA shows clearer contours, fewer false positives, and better alignment with ground truth boundaries. It performs especially well in regions with ambiguous foreground–background transitions. These results support the earlier quantitative findings and demonstrate the superior segmentation quality of the MAA for histopathological image analysis.

### 4.4. Ablation Experimental Results

To evaluate the individual contributions of the MAF module and the AR module, we carried out some ablation experiments. Three experimental configurations were implemented to systematically investigate the performance impact of each module by incrementally incorporating them into the model.

- Baseline Model: A conventional Vision Transformer (ViT) was used as the backbone for the CAM generation stage. The generated CAMs were directly used as pseudo-labels for the segmentation stage. Neither the MAF nor AR module was involved in the baseline model.
- MAF-only Model: The MAF module replaced the standard transformer in the CAM generation stage. It hierarchically fused the attention maps from the third, fifth, and sixth layers to generate the pseudo-labels, which were directly fed into the segmentation stage without the affinity-based refinement.
- Full MAA Model: Such a model configuration represented the full implementation of our proposed MAA framework. Extending the MAF-only model, the AR module was incorporated to enhance the quality of the initial pseudo-labels before they were utilized in the segmentation stage.

Table 3 presents the quantitative results measured in terms of the IoU and Dice coefficient. The baseline model achieved an IoU of 77.35% and a Dice score of 87.34%. The MAF module alone boosted the performance up to 80.85% in IoU and 89.41% in Dice score. Such a significant improvement demonstrated the effectiveness of hierarchical multi-scale attention for capturing spatial and semantic features, particularly in gland localization and contour delineation.

Powered by the AR module, the entire MAA (MAF + AR) model attained 81.99% in IoU and 90.10% in Dice score, which indicated an increment of 1.14% in IoU and 0.69% in Dice score over the MAF-only model. Such quantitative improvements confirmed that the affinity-based refinement mechanism could assist to suppress noise in pseudo-labels and enhance the edge consistency, even under the challenging conditions of high foreground–background similarity.

Both the MAF and AR modules contribute positively in the gland segmentation tasks. The MAF module provided the primary performance improvement through the additional representation learning during CAM generation. The AR module delivered additional benefits by suppressing noise and refining boundary delineation. The experimental evidence confirmed the essential role and practical effectiveness of the MAF and AR modules in the weakly supervised gland segmentation framework.

## 5. Discussion

In the present study, the proposed MAA method is a two-stage framework, which integrates two innovative components to ameliorate the global semantic understanding and fine-grained structural refinement with only image-level labels. The MAF module exploits hierarchical features from multiple Transformer layers to enhance the semantic quality of initial class activation maps. The AR module further refines the pseudo-labels by learning the semantic affinities and enforcing local inter-pixel consistency. The refined pseudo-labels then guide the training of the segmentation network, leading to excellent performance on the GlaS dataset. The results of ablation experiments confirmed that the sole contribution of each module to the improvements in segmentation performance.

Performance comparisons with other prevailing weakly supervised segmentation methods further highlighted the advantages of the MAA. It outperformed the existing CAM-based methods such as SEAM, Adv-CAM, and SC-CAM, which mainly relied on the one-stage convolutional backbones. The MAA achieved informative region activation and improved boundary localization. Compared with the two-stage OEEM framework for weakly supervised gland segmentation, the MAA enhanced pseudo-label quality by exploring the inter-pixel relationships. Visualization results also showed that MAA can outline cleaner boundaries and provide more structurally consistent segmentation outputs.

Despite the above encouraging results, the MAA framework exhibits several limitations. First, based on a fixed selection of Transformer layers for feature fusion, the MAF module is limited to adapt optimally to diverse tissue types. Second, the AR module assumes that semantic affinity can be reliably inferred from noisy pseudo-labels, which limits the flexibility of the MAA model to the data sets with variety patterns. Moreover, the MAA framework currently only supports the pixel-wise segmentation, and its applicability to more complex clinical tasks has not been studied thoroughly.

Future research may focus on several directions to further enhance the effectiveness and applicability of the MAA framework. At the module level, one promising direction is to incorporate a feedback mechanism from the second-stage segmentation network to iteratively refine the pseudo-labels generated by the AR module, which could further improve label quality and segmentation performance. Another important aspect at the module level is to perform a systematic robustness evaluation of the AR module under varying levels of noise in the initial CAMs, which would provide a deeper understanding of its stability and reliability in practical scenarios. At the network level, future studies could explore adaptive attention fusion mechanisms that dynamically assign weights to different semantic levels based on input characteristics. This would enable the model to better capture context-specific features and improve generalization on diverse histopathological datasets. Investigating the performance of the proposed MAF and AR modules with more modern segmentation backbones is also an interesting direction, which could potentially enhance feature representation and overall segmentation performance. At the application level, incorporating domain-specific priors, such as morphological constraints or tissue texture, may help accelerate learning and suppress anatomically implausible predictions. Moreover, the MAA framework could be extended to multi-class or instance-level segmentation, particularly for the scenarios that involve overlapping structures or heterogeneous tissue regions.

The present study highlights the potential of integrating attention-based feature extraction with affinity-guided label refinement for weakly supervised medical image analysis. The encouraging results suggested a viable solution that can alleviate annotation burdens and retain a high segmentation accuracy. Future research should investigate the generalizability of transformer-driven architectures and inter-pixel semantic modeling for a variety of medical imaging tasks, which could expand the clinical applicability of weakly supervised methods.

## 6. Conclusions

Segmentation of glands in histopathological images plays a pivotal role in the diagnosis and grading of colorectal cancer. However, most of popular deep-learning methods rely on dense pixel-level annotations, which are labor-intensive. It is a challenge to preserve the segmentation accuracy when reducing the annotation labels during model training in medical image analysis. In the present study, the MAA model that only requires image-level labels for training is proposed as a novel two-stage weakly supervised segmentation framework.

The MAA incorporates two core components to improve pseudo-label quality and segmentation precision. The MAF module extracts and aggregates the hierarchical Transformer features, which assists the model to capture rich semantic context and produce the comprehensive class activation maps. The AR module leverages inter-pixel semantic consistency to suppress noisy activations and refine boundary accuracy. Experimental results on the GlaS dataset confirmed the effectiveness of MAA, which outperformed several baseline approaches, including general-purpose CAM-based models and a domain-specific two-stage framework. Such results underscore the benefits derived from the integration of global attention mechanisms with local affinity learning in weakly supervised segmentation.

Despite its advantages, the current framework still has some limitations. The fixed fusion strategy in the MAA module may limit generalization to diverse tissue structures, and the method currently supports only binary segmentation. Future research may explore adaptive fusion mechanisms, incorporate domain-specific priors such as gland morphology and texture, and extend the framework to multi-class and instance-level tasks. Based on the present study and the promising future directions, we expect to provide a more solid scientific basis and practical support for the development of annotation-efficient medical image segmentation methods in clinical applications.

## Figures and Tables

**Table 1:** Details of the whole-slide images in the GlaS dataset [27].

Histologic Grade	Number of Images (Width × Height in Pixels)
Benign	$74\left\{\begin{array}{cc} \;2\; & \left(574\times 433\right)\\ \;5\; & \left(589\times 453\right)\\ \;67\; & \left(775\times 522\right) \end{array}\right.$
Malignant	$91\left\{\begin{array}{cc} \;2\; & \left(574\times 433\right)\\ \;5\; & \left(589\times 453\right)\\ \;84\; & \left(775\times 522\right) \end{array}\right.$

**Table 2:** Performance comparison between the MAA framework and other weakly supervised methods. The IoU and Dice results are listed in terms of mean ± standard deviation in percentage.

Method	Framework Type	Backbone	IoU (%)	Dice (%)
SEAM [23]	One-stage	ResNet-38	66.11 ± 3.26	79.59 ± 4.88
Adv-CAM [24]	One-stage	ResNet-101	68.54 ± 3.36	81.33 ± 5.26
SC-CAM [25]	One-stage	ResNet-50	71.52 ± 3.50	83.40 ± 5.36
OEEM [26]	Two-stage	ResNet-50 + PSPNet	77.56 ± 3.12	87.36 ± 4.39
MAA (Ours)	Two-stage	Transformer + PSPNet	81.99 ± 2.26	90.10 ± 3.31

**Table 3:** Contributions of the MAF and AR modules to the gland segmentation performance in the ablation experiments. The sign of “–” indicates the module was excluded, and “✓” indicates the module was involved. The IoU and Dice results are listed in terms of mean ± standard deviation in percentage.

Method	MAF	AR	IoU (%)	Dice (%)
Baseline	–	–	77.35 ± 3.19	87.22 ± 4.42
MAF-only	✓	–	80.85 ± 2.60	89.41 ± 3.53
MAA (Ours)	✓	✓	81.99 ± 2.26	90.10 ± 3.31

## Data Availability

The data used in this study are obtained from the GlaS dataset of the MICCAI 2015 Histology Image Challenge [27], which is openly available at https://www.kaggle.com/datasets/sani84/glasmiccai2015-gland-segmentation/ (accessed on 17 September 2025).

## Abbreviations

The following abbreviations are used in this manuscript:

AR	Affinity Refinement
ASPP	Atrous Spatial Pyramid Pooling
CAM	Class Activation Map
CNN	Convolutional Neural Network
FCN	Fully Convolutional Network
GlaS	Gland Segmentation in Colon Histology Images Challenge Contest
H & E	Hematoxylin and Eosin
MAA	Combination of Multi-Level Attention and Affinity
MAF	Multi-Level Attention Fusion
MiT	Mix Transformer
MLP	Multi-Layer Perceptron
IoU	Intersection over Union
OEEM	Online Easy Example Mining
PAR	Pixel-Adaptive Refinement module
PSPNet	Pyramid Scene Parsing Network
ResNet	Residual Networks
SEAM	Self-Supervised Equivariant Attention Mechanism
SGD	Stochastic Gradient Descent
ViT	Vision Transformer
WSIS	Weakly Supervised Image Segmentation

## References

[1] Kuipers E., Rösch T., Bretthauer M. (2013). Colorectal cancer screening – optimizing current strategies and new directions. Nat. Rev. Clin. Oncol..

[2] Rathore S., Hussain M., Ali A., Khan A. (2013). A recent survey on colon cancer detection techniques. IEEE/ACM Trans. Comput. Biol. Bioinf..

[3] Lang-Schwarz C., Melcher B., Hausmeier F., Schneider-Fuchs A., Lang-Schwarz K., Krugmann J., Vieth M., Sterlacci W. (2019). Budding, tumor-infiltrating lymphocytes, gland formation: Scoring leads to new prognostic groups in World Health Organization low-grade colorectal cancer with impact on survival. Hum. Pathol..

[4] Pacal I., Karaboga D., Basturk A., Akay B., Nalbantoglu U. (2020). A comprehensive review of deep learning in colon cancer. Comput. Biol. Med..

[5] Hamida A.B., Devanne M., Weber J., Truntzer C., Derangere V., Ghiringhelli F., Forestier G., Wemmert C. (2021). Deep learning for colon cancer histopathological images analysis. Comput. Biol. Med..

[6] Liu X., Qu L., Xie Z., Zhao J., Shi Y., Song Z. (2024). Towards more precise automatic analysis: A systematic review of deep learning-based multi-organ segmentation. Biomed. Eng. Online.

[7] Rastogi P., Khanna K., Singh V. (2022). Gland segmentation in colorectal cancer histopathological images using U-net inspired convolutional network. Neural Comput. Appl..

[8] Tharwat M., Sakr N.A., El-Sappagh S., Soliman H., Kwak K.S., Elmogy M. (2022). Colon cancer diagnosis based on machine learning and deep learning: Modalities and analysis techniques. Sensors.

[9] Fu Y., Lei Y., Wang T., Curran W.J., Liu T., Yang X. (2021). A review of deep learning based methods for medical image multi-organ segmentation. Phys. Medica.

[10] Rathore S., Iftikhar M.A., Chaddad A., Niazi T., Karasic T., Bilello M. (2019). Segmentation and grade prediction of colon cancer digital pathology images across multiple institutions. Cancers.

[11] Siddique N., Paheding S., Elkin C.P., Devabhaktuni V. (2021). U-Net and its variants for medical image segmentation: A review of theory and applications. IEEE Access.

[12] Chen H., Qi X.J., Yu L.Q., Dou Q., Qin J., Heng P.A. (2017). DCAN: Deep contour-aware networks for object instance segmentation from histology images. Med. Image Anal..

[13] Hatamizadeh A., Tang Y., Nath V., Yang D., Myronenko A., Landman B., Roth H.R., Xu D. Unetr: Transformers for 3d medical image segmentation. Proceedings of the IEEE/CVF Winter Conference on Applications of Computer Vision.

[14] Liang Q., Nan Y., Coppola G., Zou K., Sun W., Zhang D., Wang Y., Yu G. (2019). Weakly supervised biomedical image segmentation by reiterative learning. IEEE J. Biomed. Health. Inf..

[15] Lin D., Dai J., Jia J., He K., Sun J. Scribblesup: Scribble-supervised convolutional networks for semantic segmentation. Proceedings of the 2016 IEEE Conference on Computer Vision and Pattern Recognition (CVPR 2016).

[16] Dai J., He K., Sun J. Boxsup: Exploiting bounding boxes to supervise convolutional networks for semantic segmentation. Proceedings of the 2015 IEEE Conference on Computer Vision (ICCV 2015).

[17] Maninis K.K., Caelles S., Pont-Tuset J., Van Gool L. Deep extreme cut: From extreme points to object segmentation. Proceedings of the 2018 IEEE Conference on Computer Vision and Pattern Recognition (CVPR 2018).

[18] Ahn J., Kwak S. Learning pixel-level semantic affinity with image-level supervision for weakly supervised semantic segmentation. Proceedings of the 2018 IEEE Conference on Computer Vision and Pattern Recognition (CVPR 2018).

[19] Zhou B., Khosla A., Lapedriza A., Oliva A., Torralba A. Learning deep features for discriminative localization. Proceedings of the 2016 IEEE Conference on Computer Vision and Pattern Recognition (CVPR 2016).

[20] Wei Y., Feng J., Liang X., Cheng M.M., Zhao Y., Yan S. Object region mining with adversarial erasing: A simple classification to semantic segmentation approach. Proceedings of the 2017 IEEE Conference on Computer Vision and Pattern Recognition (CVPR 2017).

[21] Jiang P.T., Hou Q., Cao Y., Cheng M.M., Wei Y., Xiong H. Integral object mining via online attention accumulation. Proceedings of the 2019 IEEE/CVF International Conference on Computer Vision (ICCV 2019).

[22] Lin T.Y., Maire M., Belongie S., Hays J., Perona P., Ramanan D., Dollar P., Zitnick C.L. Microsoft COCO: Common objects in context. Proceedings of the 2014 European Conference on Computer Vision (ECCV 2014).

[23] Wang Y., Zhang J., Kan M., Shan S., Chen X. Self-supervised equivariant attention mechanism for weakly supervised semantic segmentation. Proceedings of the 2020 IEEE/CVF Conference on Computer Vision and Pattern Recognition (CVPR 2020).

[24] Lee J., Kim E., Yoon S. Anti-adversarially manipulated attributions for weakly and semi-supervised semantic segmentation. Proceedings of the 2021 IEEE Conference on Computer Vision and Pattern Recognition (CVPR 2021).

[25] Chang Y.T., Wang Q., Hung W.C., Piramuthu R., Tsai Y.H., Yang M.H. Weakly-supervised semantic segmentation via sub-category exploration. Proceedings of the 2020 IEEE Conference on Computer Vision and Pattern Recognition (CVPR 2020).

[26] Li Y., Yu Y., Zou Y., Xiang T., Li X. Online easy example mining for weakly-supervised gland segmentation from histology images. Proceedings of the 25th International Conference on Medical Image Computing and Computer Assisted Intervention (MICCAI 2022).

[27] Sirinukunwattana K., Pluim J.P.W., Chen H., Qi X., Heng P.A., Guo Y.B., Wang L.Y., Matuszewski B.J., Bruni E., Sanchez U. (2017). Gland segmentation in colon histology images: The glas challenge contest. Med. Image Anal..

[28] Xie E., Wang W., Yu Z., Anandkumar A., Alvarez J.M., Luo P. (2021). SegFormer: Simple and efficient design for semantic segmentation with transformers. Adv. Neural Inf. Process. Syst..

[29] Ru L., Zhan Y., Yu B., Du B. Learning affinity from attention: End-to-end weakly-supervised semantic segmentation with transformers. Proceedings of the 2022 IEEE/CVF Conference on Computer Vision and Pattern Recognition (CVPR 2022).

[30] Vernaza P., Chandraker M. Learning random-walk label propagation for weakly-supervised semantic segmentation. Proceedings of the 2017 IEEE Conference on Computer Vision and Pattern Recognition (CVPR 2017).

[31] Zhao H., Shi J., Qi X., Wang X., Jia J. Pyramid scene parsing network. Proceedings of the 2017 IEEE Conference on Computer Vision and Pattern Recognition (CVPR 2017).

[32] Wang Z., Wang E., Zhu Y. (2020). Image segmentation evaluation: A survey of methods. Artif. Intell. Rev..

[33] Dice L.R. (1945). Measures of the amount of ecologic association between species. Ecology.

